# Proceedings of the 2015 Meeting of the Australasian Section of the American Oil Chemists’ Society (AAOCS)

**DOI:** 10.3390/nu7125518

**Published:** 2015-12-02

**Authors:** Matthew R. Miller, Tim D. Nalder, Jacqui L. Adcock

**Affiliations:** 1 Cawthron Institute, Nelson 7010, New Zealand; 2 Centre for Chemistry and Biotechnology, School of Life and Environmental Sciences, Waurn Ponds Campus, Deakin University, Geelong 3220, Australia; tim.nalder@deakin.edu.au (T.D.N.); jadcock@deakin.edu.au (J.L.A.); 3 Marine Products Team, The New Zealand Institute for Plant & Food Research Ltd., 300 Wakefield Quay, Nelson 7010, New Zealand

## 1. Preface

The Australasian section of the American Oil Chemists’ Society (AAOCS) held their 9th biennial meeting in Geelong, Australia from 9 to 11 September 2015. Over 100 scientists, researchers and industry representatives gathered for the conference and two industry focused workshops. The conference theme was “looking back, thinking forward” which reflected on the history of the science and industry while also predicting future trends and issues. Two pre-conference workshops were held on spreads and margarine formulation, and infant formula lipids. The AAOCS Award for Scientific Excellence in Lipid Research was awarded to Prof. Andrew Sinclair. Prof. Sinclair is an Emeritus Professor of Nutrition Science within the School of Medicine at Deakin University, Geelong, Australia. He has been a significant contributor to the AOCS and the Australasian section of AOCS since 1995, as a chairperson, committee member and as an editorial board member.

Day one of the conference was hosted jointly with the Omega 3 Centre and the opening address was given by Prof. Thomas Sanders from King’s College, London, on dietary fat in relation to cardiovascular health. This kicked off a very interesting session on the role of omega-3 long chain polyunsaturated fatty acids (LCPUFA) and other dietary fats in heart health. Prof. Manny Noakes, The Commonwealth Scientific and Industrial Research Organisation (CSIRO), showed that omega-3 LCPUFA supplements have neither a beneficial nor adverse effect demonstrated in primary or secondary prevention of coronary heart disease (CHD) (Nestel, P., *et al*. *Heart Lung Circ*. 2015, *24*, 769). The evidence continues to be positive for the role of omega-3 LCPUFA in the treatment of hypertriglyceridaemia and beneficial effects in preventing heart failure. Higher fish intake is associated with lower incident rates of heart failure in addition to lower sudden cardiac death, stroke and myocardial infarction. Prof. Peter McLennan from the University of Wollongong, examined the pitfalls in the use of randomised controlled trials for fish oil studies and concluded that understanding the pleiotropic mechanisms of action of omega-3 LCPUFA and better interpretation of the epidemiology must be used to improve randomised controlled trial design and analysis in order to resolve the apparent contradictory effects of omega-3 LCPUFA supplements and eating fish.

Dr Peter Nichols from CSIRO talked about long-chain omega-3 oils: sources, ingredient quality and methods of analysis particularly in the wake of a recent controversial publication (Albert, B.B., *et al*. *Sci. Rep*. 2015, *5*, 7928). Dr Nichols showed that in follow up work on Australian and NZ oil supplements by different organisations, retesting of marine oil products gave satisfactory results for all products in relation to PV and content of omega-3 fatty acids, in contradiction to the previous report. Dr Laurence Eyres took us through the history of the yellow fats market in Australasia, with audible gasps from many of the attendees at the content of *trans* fats in historic margarines. Prof. Colin Barrow from Deakin University looked at future biotechnological possibilities, such as endogenous omega-3 derived bioactive compounds like resolvins and protectins, as well as the use of novel enzymes in marine oil processing. Assoc. Prof. Nils Hoem, Aker BioMarine, posed the question: “Is the classical bioavailability concept suitable to describe the systemic uptake of endogenous lipids?” He concluded that the term digestibility may be more appropriate, although it may be technically demanding. The CSIRO Omega Team was well represented at the meeting and entertained with their world leading technologies in developing new oils from land plants. Team leader, Dr Surinder Singh, showed that DHA canola has moved from the lab and into the field, and the target release date for novel omega-3 enriched plant oils will be in 2018/19. We also want to congratulate Surinder on being named one of Nature Biotech’s top 20 translational researchers of 2014.

A hot topic session on olive oil was a highlight of the conference. The continuing issues with adulteration of virgin olive oils, global challenges and opportunities, quality, standards, nutrition and processing were all covered in a series of keynote presentations by Paul Miller (Australian Olive Association), Dr Rod Mailer (Australian Oils Research), Claudia Guillaume (Modern Olives) and Dr Leandro Ravetti (Modern Olives).

A goal of the Australasian section of AOCS is helping to develop future researchers and industry leaders. We had a number of student oral and poster presentations, which were of very high quality. Tim Walsh from Deakin University and Courtney Davis from University of South Australia shared the Bryce Bell student prize for best oral communication. Walsh’s work looked at the production of resolvins and protectins with plant derived enzymes. These products have potential for use as therapeutic agents against chronic inflammatory diseases, or as dietary supplements to help prevent such diseases. Davis’s work looked at olive oil in the Mediterranean diet and changes in erythrocyte fatty acids translating to a more favourable heart health profile. The Rod Mailer student poster prize was awarded to Jessica Learey from Deakin University who developed new methods (chemiluminescence and electron paramagnetic resonance) to determine oxidation in pet foods. The AAOCS is very supportive of all our students and will contribute to students to travel to the AOCS events in the USA in the future.

## 2. Summary of Scientific Presentations

### 2.1. Dietary Fat in Relation to Cardiovascular Health—Looking Back and Looking Forward

#### Sanders, T.A.B.

For more than 30 years, dietary advice for the prevention of cardiovascular disease has recommended that fat and saturated fatty acids (SFA) supply no more 30%–35% and 10% of the dietary energy. However, prospective studies have failed to link saturated fat intake to increased risk of CVD and a recent meta-analysis of the trial concluded that the recommendations should never have been made. However, elevated serum total cholesterol (TC) is a key factor for coronary heart disease (CHD), along with smoking and high blood pressure. Replacing saturated fatty acids with unsaturated fatty acids contributes to lowering TC but medium chain and stearic acid have no effect. Furthermore, these dietary effects on TC are modest compared with statins. Nevertheless, the increased use of unhydrogenated vegetable oils and a reduction in the use of animal fats, have resulted in reductions in SFA from 20% to 12% energy which has been accompanied by small falls in average serum cholesterol. For reasons that are uncertain, the incidence of CVD is falling strongly in many developed economies. The epidemiological evidence linking trans fatty acids to CHD is stronger but the evidence suggest that polyunsaturated fatty acids both from fish as well as those from vegetable oils are associated with lower risk. The evidence base for setting an upper limit of 35% energy from fat is currently lacking and the Mediterranean dietary pattern (up to 46% energy fat) is associated with a lowered risk of CHD compared to the standard low fat advice.

### 2.2. Omega-3 Nutrition—Fish versus Supplementation

#### Noakes, M.

A review on omega-3 long-chain polyunsaturated fatty acids (LCPUFA) was undertaken in 2014 and included all literature published between 1 January 2007 and 31 August 2013 (Nestel, P., *et al.* *Heart Lung Circ.* 2015, *24*, 769–779).

The recommendation from these data is that there has been no further evidence to recommend supplementation with omega-3 LCPUFA for primary or secondary prevention of heart disease. However, intake of fish was consistently associated with lower risk of heart disease and stroke. Higher fish intake was associated with lower incident rates of heart failure in addition to lower sudden cardiac death, stroke and myocardial infarction. The evidence for stroke prevention was stronger for fish consumption than for omega-3 LCPUFA supplements.

LCPUFA benefits the treatment of hypertriglyceridaemia, heart failure, atrial fibrillation and hypertension. Stiffness of large arteries due to aging or disease associates with CVD events. Importantly both arterial stiffness and endothelial dysfunction are ameliorated through LCPUFA consumption. Fish oil and both EPA and DHA supplementation helps reduce arterial stiffness. Non-cardiovascular focussed studies have shown benefits of LCPUFA supplements for patients with depression, ADHD and inattention symptoms. LCPUFA also appear to provide adjunctive treatment for joint pain associated with rheumatoid arthritis, inflammatory bowel disease, and dysmenorrhea. The Heart Foundation currently recommends that all Australian's should aim to include two to three serves of fish (including oily fish) per week as part of a heart healthy eating pattern. This amount of fish provides between 250 and 500 mg per day of combined docosahexaenoic acid (DHA) and eicosapentaenoic acid (EPA).

### 2.3. To Fish or Supplement: Is that the Question for Cardiovascular Risk Reduction?

#### McLennan, P.L.; Pepe, S.

Regular fish consumption is associated with low cardiovascular disease risk. However, randomised control trials (RCT) of fish oil supplements produce variable results, interpreted to not support either beneficial or adverse effects of long chain n-3 polyunsaturated fatty acids (LC n-3 PUFA) eicosapentaenoic acid (EPA) and docosahexaenoic acid (DHA) in the treatment or prevention of cardiovascular disease (Nestel, P., *et al.* *Heart Lung Circ.* 2015, *24*, 769–779). This review considers issues key to understanding the contradiction. (1) When is a placebo not a control? Most RCT report no exclusion criteria for fish eaters and even fewer analyse n-3 PUFA status. Even after exclusions, trials report overlap of tissue n-3 PUFA status in control and treated groups; (2) Cardiac effects (sudden death, heart failure prevention) require myocardial membrane incorporation of DHA. The LC n-3 PUFA intake in cohort studies derives from seafood providing DHA > EPA, whereas most RCT give supplements rich in EPA, low in DHA; (3) Nutritional and therapeutic dose effects of LC n-3 PUFA are defined by different mechanisms. Direct cardiac effects on heart rate, heart failure and sudden death occur at lower doses than influence classical coronary artery disease risk factors. RCT designs combine cardiac and arterial disease populations and composite endpoints, which propose a common substrate and assume a common mechanism of action. Conclusion: Understanding the pleiotropic mechanisms of action of LC n-3 PUFA and better interpretation of the epidemiology must be used to improve RCT design and analysis in order to resolve the apparent contradictory effects of LC n-3 PUFA supplements and eating fish.

### 2.4. Bioavailability of Long Chain Omega 3 Fatty Acids is Influenced by the Fish Oil Feeding Strategy (Daily vs. Weekly)

#### Ghasemifard, S.; Sinclair, A.J.; Lewandowski, P.; Turchini, G.M.

The recommendations on the intake of long chain omega-3 polyunsaturated fatty acids (n-3 LC-PUFAs) vary from eating fish (“once to twice of oily fish per week”) to consuming specified amounts of EPA and DHA (“250–500 mg per day”). It is not known if there is a difference in the uptake/bioavailability between consuming fish once or twice per week or taking supplements every day. In this study, the bioavailability of a daily dose of n-3 LC-PUFA (Constant treatment), representing supplements, *versus* a large weekly dose of n-3 LC-PUFA (Spike treatment), representing consuming once or twice per week, was assessed. Six-week old healthy male Sprague-Dawley rats were fed either a Constant treatment, a Spike treatment or Control treatment (no n-3 LC-PUFA), for six weeks. The whole body, tissues and collected faeces were analysed for fatty acid content. The main fate of EPA was β-oxidation, explaining its low accumulation in tissues. DPA was the next most deposited n-3 LC-PUFA in tissues except the liver where EPA levels were significantly greater than DPA level. Treatments significantly affected the metabolic fate of dietary n-3 LC-PUFA: in rats consuming the Spike treatment, n-3 LC-PUFA were deposited to a greater extent and β-oxidized less compared with the Constant treatment. These findings suggests that a large dose of n-3 LC-PUFA once per week is more effective in increasing whole body n-3 LC-PUFA content compared with a smaller dose delivered daily.

### 2.5. Long-Chain Omega-3 Oils: Sources, Ingredient Quality and Methods of Analysis

#### Nichols, P.D.

Considerable evidence exists on the health benefits of long-chain (≥C20), LC omega-3 oils, in particular EPA (eicosapentaenoic acid, 20:5w3) and DHA (docosahexaenoic acid, 22:6w3). Notwithstanding, current topics of interest in this still growing research area and its associated markets include: the diminishing fish oil resource against expanding market demand, competition for the resource (e.g., aquafeed *versus* higher value supplements and pharmaceuticals), decreasing product quality in farmed seafood in terms of LC omega-3 content, the different role of shorter chain and LC omega-3 and resulting consumer confusion, the resulting need for new, sustainable sources of LC omega-3, the recent and at times on-going negative media (prostate, heart health) towards LC omega-3, the form of LC omega-3 (triacylglycerol, ethyl ester, polar lipid), the specific source (fish, krill, squid), and analytical issues with specific emphasis on accurate measurement of LC omega-3 and oil quality. 2015 has seen linking of alleged quality issues of LC omega-3 with various negative media. An advisory need for progressing and/or responding to developments in many of these areas sees considerable input in North America and Europe from the international body GOED (global organization for EPA and DHA). In Australia and New Zealand, the Omega-3 Centre, including its scientific advisors and committee, play a lead advisory role with LC omega-3 oils. A number of the topics and issues raised above will be discussed with an emphasis on mainly Australian developments and case studies, including with focus on methods of analysis and concerns by industry on recent scientific findings.

### 2.6. Desorption Electrospray Ionization (DESI) for Mapping the Intact Omega-3 Lipids in Greenshell™ Mussels

#### Miller, M.R.; Joyce, N.

Desorption electrospray ionization mass spectrometry (DESI-MS) is a powerful new semi-quantifiable analytical technique which can be employed in a diverse range of applications, including the study of drug incorporation into organs. DESI-MS is also a versatile tool for lipid chemistry of biological systems, as it enables the rapid determination of lipid structures, and the direct correlation of anatomic features with individual lipid species.

Here we report the first use of DESI-MS to map the lipid profile of Greenshell™ mussel (GSM) tissue, whereby individual intact lipids and their spatial distribution within whole mussels were determined. We identified several polar lipids in GSM, and found the profile was dominated by 16:0–22:6 phosphatidylcholine (PC) species. This lipid species was found concentrated in the GSM mantel of female mussels indicating a functional role in respiration and feeding.

This study demonstrates that lipid-mapping of GSM by DESI-MS can assist our understanding of the ways dietary lipids are utilised by this bivalve, and is expected to provide key insights into how the selective breeding, production and processing of GSM in New Zealand can be optimised.

### 2.7. Metabolic Fate (Absorption, β-Oxidation and Deposition) of Long Chain Omega 3 Fatty Acids Is Affected by Sex and by the Oil Source (Krill Oil or Fish Oil) in the Rat

#### Ghasemifard, S.; Hermon, K.; Turchini, G.M.; Sinclair, A.J.

The effects of krill oil as an alternative source of long chain omega 3 fatty acids (n-3 LC-PUFA) have been investigated recently. There are conflicting results from the few available studies comparing fish oil and krill oil. The aim of this study was to compare the bioavailability and metabolic fate (absorption, β-oxidation and tissue deposition) of omega 3 fatty acids originating from krill oil (phospholipid-rich) or fish oil (triglyceride-rich) in rats of both sexes using the Whole Body Fatty Acid Balance Method. Sprague-Dawley rats (36 male, 36 female) were randomly assigned to have either a krill oil diet (EPA + DHA + DPA = 1.38 mg/g of diet) or a fish oil diet (EPA + DHA + DPA = 1.61 mg/g of diet) to constant ration for six weeks. The faeces, whole body and individual tissues were analysed for fatty acid content.

Absorption of fatty acids was significantly greater in female rats, and was only minimally affected by the oil type. It was estimated that most of EPA (>90%) and more than half of DHA (>60%) were β-oxidised in both diet groups. Most of the DPA was β-oxidised (57% and 67% for female and male rats, respectively) in the fish oil group; however, for the krill oil group, the majority of DPA was deposited (82%–83%). There was a significantly greater deposition of DPA and DHA in rats fed krill oil compared with those fed fish oil, not due to a difference in bioavailability (absorption), but rather to a difference in metabolic fate (anabolism *vs*. catabolism).

### 2.8. Effect of a Mediterranean Diet on the Omega-3 Index (O3I) in Older Australians; Results from the Mediterranean Diet (MedDiet) for Cognition and Cardiovascular Health in the Elderly (MedLey) Trial

#### Murphy, K.J.; Davis, C.R.; Bryan, J.; Wilson, C.; Hodgson, J.M.

The Mediterranean diet (MedDiet), reported to have cardiovascular health benefits, consists of a variety of foods including fruit and vegetables, wholegrain cereals, legumes, nuts, red wine, olive oil, fish, poultry and red meat delivering valuable nutrients like fibre, polyphenols and omega-3 fatty acids. We sought to evaluate the health benefits of a Mediterranean diet on cardiovascular health outcomes in older Australians in the MedLey trial. Participants were randomly allocated either a MedDiet (*n* = 70) or continue their habitual diet (HabDiet) (*n* = 67) for six months. A range of cardiovascular outcomes were measured at baseline and three and six months including the O3I reported here.

Fish intake increased in the MedDiet group from 33 ± 41 g/day (Mean ± SD) at baseline to 60 ± 45 g/day and 72 ± 45 g/day at 2 and 4 months, respectively (*p* < 0.01), with no change in the HabDiet group (47 ± 49 g/day, 38 ± 50 g/day, 34 ± 47 g/day at baseline and two and four months, respectively). The O3I (%DHA + EPA) of all participants prior to the intervention was 7.5% (SD 2.2), which is heading towards the desirable target for protection from death from coronary heart disease. The O3I increased from 7.6 ± 0.3 (mean ± SEM) to 7.9 ± 0.2 and 8.0 ± 0.2 in the MedDiet group compared with 7.3 ± 0.3 to 7.4 ± 0.3 and 7.6 ± 0.3 the HabDiet group (*p* = 0.025) from baseline to three and six months, respectively. There was an inverse relationship between the O3I (β = −0.257 *p* < 0.0001) and DHA (β = −0.205 *p* < 0.003) and blood triglycerides in the MedDiet group, but not the HabDiet.

Compared with HabDiet, the MedDiet group (with an already moderate-high protective baseline O3I), significantly increased their fish intake according to study dietary prescriptions resulting in a further significant increase in the O3I which was negatively related to blood triglycerides.

### 2.9. Is the Classical Bioavailability Concept Suitable to Describe the Systemic Uptake of Endogenous Lipids?

#### Hoem, N.

The term “bioavailability” (BA) is commonly used to describe how different formulations of bio-active molecules may influence the completeness of systemic uptake. The concepts and the methods by which it is performed and analysed were developed in pharmaceutical research, especially in the area of generics.

Increasingly the use of BA analysis has migrated into other areas such as uptake of endogenous substances. The concept is frequently used in scientific publications dealing with the uptake, distribution, metabolism and elimination (ADME) of lipids. Thus, in the literature as well as in marketing material on different forms/formulations of EPA and DHA containing lipids, BA claims have become common.

Unfortunately, during its migration from pharmaceutical development into other areas, the loss of regulatory rigorousness has created more confusion than clarification to its intended purpose.

With specific reference to EPA and DHA, this presentation will evaluate past and present use and outline the inherent conceptual and procedural limitations of such use of the BA concept.

### 2.10. Bioavailability of Microencapsulated Fish Oil Powders

#### Augustin, M.A.; Sanguansri, L.; Lockett, T.; Stonehouse, W.

Omega-3 (ω3) long chain polyunsaturated fatty acids (LC-PUFA), derived from marine sources, are recognised for their health benefits. Due to human’s limited capacity to synthesize ω3 LC-PUFA its consumption through diet is important. The Australian population may be at risk of ω3 LC-PUFA deficiency. Foods enrichment with ω3 LC-PUFA could contribute significantly to achieving recommended intakes. Microencapsulated fish oil ingredients are protected against oxidation and may be incorporated into foods with acceptable sensory properties, but it is also essential to examine the bioavailability of ω3 LC-PUFA from these foods. Microencapsulated fish oil-in-water emulsions formulated with heated mixtures of protein and carbohydrates may be converted into shelf-stable powdered ingredients (Sanguansri, L., *et al*. Patent WO200174175, 11 October 2001). Human trials with microencapsulated fish oil powder enriched foods provide the evidence that microencapsulation of the oils within heated protein-carbohydrate matrices does not compromise the bioavailability of ω3 LC-PUFA. A study with human ileostomates showed that <1% of the delivered ω3 LC-PUFA remained in the ileal effluent irrespective of whether the subject consumed a fish oil capsule or various food products (orange juice, yoghurt or cereal bar) enriched with microencapsulated fish oil. Clinical trials with healthy individuals confirmed the human bioequivalence for microencapsulated fish oil powder delivered in a flavoured milk compared to fish oil gelatin capsules taken with flavoured milk, as assessed by plasma fatty acid levels after ingestion. These results present convincing evidence that foods enriched with microencapsulated oil powders within heated protein-carbohydrate matrices may be an effective and safe strategy for delivering bio-available of ω3 LC-PUFA.

### 2.11. Microencapsulated Omega-3 Oil Powder Performance in Extrusion and Tabletting Application

#### Sanguansri, L.; Ying, D.; Augustin, M.A.

There is interest in the development of new product delivery formats for omega-3 ingredients to offer more choices for consumers and to increase their omega-3 fatty acids intake. There is no shortage of omega-3 ingredients in the market available for a whole range of products, but much less in extruded products and tablet supplements. Extruded food products (cereals and snacks) and tablets (nutritional supplements) are two product delivery formats for omega-3 oils with huge potential market, but successful product development in this product categories remain challenging due to the combination of high temperature and shear process used in extrusion cooking, and the high compression force used in tabletting. Omega-3 oil powder ingredients made by spray drying oil-in-water emulsions encapsulated within a heated protein-carbohydrate matrix using MicroMAX technology have low free oil and long shelf life (≥2 years). We investigated the effect of formulation, oil loading, process conditions and order of processing on the (i) oxidative stability of MicroMAX encapsulated omega-3 oil powder and (ii) resistance to oil leakage during extrusion and compression. In extruded product applications, the oxidative stability of extruded products using MicroMAX encapsulated omega-3 oil powder increased three-fold compared to direct oil addition. In tabletting applications, the addition of pectin to primary omega-3 oil emulsion stabilised by heated milk protein-carbohydrate mixture before spray drying, or embedding the spray dried omega-3 oil powder in a secondary gelatine matrix before extrusion and drying into granules significantly improved resistance of powder to oil leakage during compression into tablets.

### 2.12. Microencapsulation of Tuna Oil in Gelatin-Sodium Hexametaphosphate Using Complex Coacervation

#### Wang, B.; Adhikari, B.; Barrow, C.J.

The microencapsulation of tuna oil in gelatin-sodium hexametaphosphate (SHMP) using complex coacervation was optimised for the stabilisation of omega-3 oils, for further use as a functional food ingredient. Firstly, oil stability was optimised by comparing the accelerated oxidative stability of tuna oil in the presence of various commercial antioxidants using a Rancimat. Then zeta-potential (mV), turbidity and coacervate yield (%) were measured and optimised for complex coacervation. The highest yield of complex coacervate was obtained at pH 4.7 and at a gelatin to SHMP ratio of 15:1. Multi-core microcapsules were formed when the mixed microencapsulation system was cooled to 5 °C at a rate of 12 °C/h. Crosslinking with transglutaminase followed by freeze drying resulted in a dried powder with an encapsulation efficiency of 99.82% and a payload of 52.56%. 98.56% of the oil was successfully microencapsulated and the Fourier transform infrared spectra suggested that there was no observable oxidation of the oil during microencapsulation. The accelerated oxidative stability test showed stability of the tuna oil microcapsules were more than double that of non-encapsulated oil.

### 2.13. Evolution and Extinction Amongst the Yellow Fats Market in Australasia

#### Eyres, L.

The original invention of margarine (80% fat) as a butter substitute was published as a patent in 1814 by a French Chemist Meges Mourieres. Since then the original product, based on beef tallow, has evolved into refrigerator spreadable products based on predominantly blends of hard fats and liquid vegetable oils. In UK, the original hardstock was partially hydrogenated fish oil blended with some palm oil and any liquid vegetable oil. In USA, the predominant fat was soybean oil followed by cottonseed. In Australasia, margarines took a long time to emerge as credible pleasant tasting alternatives to butter due to the draconian dairy regulation in both Australia and New Zealand. A short history of the politics and market shares will be presented. The medical lobby in both countries resulted in the launching of polyunsaturated margarine in the early 1970s. Based on hydrogenated soybean/sunflower oil, the products contained around 11%–15% *trans* fats but with <20% saturated fat. Margarine has now been replaced by spreads of varying fat contents and *trans* has been removed. Technological changes in formulations and interesterification will be discussed along with the different variations of nutritional and flavour innovations.

### 2.14. Omega-3 Biotechnology—Looking Back and Thinking Forward

#### Barrow, C.J.

Omega-3 fatty acids have a wide range of health benefits and are an important part of a healthy diet. Deficiencies in the consumption levels of these fatty acids has led to nutritional supplements and functional foods targeted at raising consumption levels. Pharmaceutical products based around omega-3 fatty acids have also been approved, with more in development, primarily for cardiovascular diseases. Increased product development has led to the development of sources other than fish oil, such as algal fermentation, transgenic plants and krill. In addition to new sources, key omega-3 fatty acids has been derivatised to provide modified bioactive compounds, some of which are endogenous like resolvins and protectins. These derivatives are being developed as pharmaceutical and nutritional products. Other omega-3 derivatives, such as conjugates, have been produced with dual functionality using both synthetic and enzymatic methods. Lipases have been shown to be versatile omega-3 processing catalysts, with their use resulting not only in new structured lipids but also the production of new lipid conjugates. Lipases have an advantage over other enzymes for biochemical synthesis in that they can tolerate organic solvents since they work at the oil-water interface, and so are able to synthesise a broad range of products. Finally, biotechnology approaches have led to new microencapsulation methods to stabilise omega-3 oils. These methods can also enable the incorporation of multiple functional ingredients into food products. In this presentation I will discuss the current state of omega-3 biotechnology, focusing on the topics discussed above.

### 2.15. Lipohydroperoxidase Activity of Soybean 15-Lipoxygenase: Implications for Biocatalysis and Biomarkers

#### Dobson, P.; Adcock, J.L.; Barrow, C.J.

The lipohydroperoxidase activity of soybean 15-lipoxygenase-1 (15-sLOX-1) with a range of LC-PUFA hydroperoxides, including those from DHA, EPA, DPA and arachidonic acid, is described. 15-sLOX-1 is a well-studied dioxygenase enzyme under aerobic conditions. However where oxygen is the limiting substrate the enzyme exhibits hydroperoxidase activity, catalysing the formation of reactive oxygen- and carbon-centred lipid radicals. In this study DHA was used as a model substrate. A complete hypoxic reaction system was established where the dioxygenase activity of 15-sLOX-1 rapidly consumed oxygen (within 0.6 min) and DHA to produce 17-hydroperoxy-DHA. We have shown the products stem from the metabolism of 17-hydroperoxy-DHA by monitoring the substrate and product profile throughout the reaction. The products were detected and characterised by a range of chromatographic and spectroscopic techniques. Major products from DHA were identified as: (4*Z*,7*Z*,10*Z*,13*Z*,15*E*-)17-keto-heptadecapentaenoic acid, two proposed regio-isomers of hydroxy-16,17-epoxy-docosapentaenoic acid, and several isomers of a C_5_H_9_-DHA branched compound. In addition the radical scavenger 4-hydroxy-TEMPO was utilised to establish the detected compounds are products of both 15-sLOX-1 and non-enzymatic pathways. The formation of these compounds has implications for the use of 15-sLOX-1 for the production of resolvin analogs and precursors, as lipohydroperoxidase activity significantly reduces the yield of the desired product. Hydro(pero)xy-fatty acids produced by 15-sLOX-1 are also commonly used as reagents without purification, therefore contamination may be likely which has previously not been considered, especially for *in vivo*/vitro studies. Finally, detection of these compounds in biological samples may serve as an indicator of oxidative stress, hypoxia and inflammation.

### 2.16. Plant Based Enzymatic Synthesis of Resolvin and Protectin Analogues

#### Walsh, T.R.; Adcock, J.L.; Barrow, C.J.

Resolvins and protectins have recently been discovered as potent mediators of inflammation. They have potential for use as treatment for chronic inflammatory diseases such as arthritis, cardiovascular disease and asthma. Biosynthesis of these molecules is believed to occur in animals through enzymes such as lipoxygenase (LOX), from the omega-3 fatty acids eicosapentaenoic acid (EPA) and docosahexaenoic acid (DHA). Plants are known to contain LOX enzymes, as well as hydroperoxide metabolising enzymes, which can further modify the products of LOX catalysed reactions. However, to date there has only been a limited number of plant based enzymes utilized to produce resolvins, protectins, or analogues of these molecules. We have measured LOX activity in several plant extracts, including potato tuber, eggplant and germinated sunflower seeds. The germinated sunflower seeds also contained hydroperoxide metabolising enzyme activity. These plant extracts were used with arachidonic acid (AA), EPA, and DHA to produce a number of mono-, di-, and trihydroxy fatty acids. The regioisomerism of the products, which is a key factor for their biological activity, has been identified by analytical methods including UV-visible spectrophotometry, HPLC, TOF-MS, GC-MS and NMR. These products have potential for use as therapeutic agents against chronic inflammatory diseases, or as dietary supplements to help prevent such diseases.

### 2.17. Long Chain Omega-3 Fatty Acids in Canola: A Journey from the Laboratory to the Field

#### Singh, S.; Petrie, J.; Devine, M.

Omega-3 long-chain (≥C20) polyunsaturated fatty acids (ω3 LC-PUFA) like EPA and DHA have critical roles in human health and development with numerous studies indicating that deficiencies in these fatty acids can increase the risk or severity of cardiovascular and inflammatory diseases in particular. These fatty acids are predominantly sourced from fish and algal oils. In order to meet the increasing demand for these oils there is an urgent need for an alternative, safe and sustainable source of EPA and DHA. Over the last 10 years, we have focused on the production of ω3 LC-PUFA in oilseeds. In particular, we have had world leading success in engineering the synthesis of DHA in oilseeds. My talk will describe the transition of DHA production in seed of our model species *Arabidopsis* through to camelina and our target crop canola. We used cutting-edge gene isolation, characterisation and screening methods to identify an optimal gene pathway for DHA production. These genes were screened in multiple species for activity before being combined into a single, eight-gene construct for DHA production. I will describe the progress that has been made in engineering DHA in plant seed. DHA levels that exceed the amount typically found in bulk fish oil have now been achieved in seed oil. We will describe gene characterisation, construct designs, transgenic plants and seed oil fatty acid profiles obtained, as well as the progress of GM canola field trials.

### 2.18. Downstream Value Capture Opportunities in the Edible Oil Industry: From Discovery to Market Impact

#### Abeywardena, M.; Sambanthamurthi, R.

The global edible oil sector has experienced steady growth in recent years as a consequence of demand from both the food and non-food sectors. For example, the 2014/15 world supply of plant-based oils has been estimated at 175 million metric tons (MMT); compare this to 100 MMT traded a decade ago. Palm oil leads the market, accounting for 40% of the global supply. The fruit (*Elaeis guineensis*) from which palm oil is extracted is a rich source of lipid-soluble antioxidants. Additionally, the milling and the extraction of oil from palm-fruit generates a large volume of liquid bio-waste currently estimated at >90 MMT per annum. A novel water-soluble antioxidant complex rich in polyphenols (oil palm phenolics; OPP) has been recovered from this waste stream. The present study focused on establishing potential physiological benefits of OPP at the pre-clinical stage using both *in vitro* and *in vivo* models. OPP demonstrated strong antioxidant activity, prevented copper-mediated LDL-oxidation and dose-dependently caused relaxation in isolated vascular tissue preparations. Contractility of gut tissue was correlated with faster transit times in the whole rat model. OPP (50–100 mg GAE/day) did not influence growth rate or final body weights, but did reduce fat deposition in diet-induced obesity models. Blood pressure lowering actions of dietary OPP were evident in several models (*p* < 0.05) and correlated with strong vasodilator responses attained following intravenous administration. OPP possesses multiple potential health benefits which position it well as a cost-effective option to meet the rising global demand for health care based on bioactives.

### 2.19. Olive Oil in 2015. Global Challenges and Opportunities—Quality, Standards and Trade

#### Miller, P.

In 2015, the global olive oil industry faces fundamental challenges that threaten the viability of olive oil producers everywhere. While there is increasing market demand for products labelled as higher quality, this is not matched by the true quality of the supply or by possible advances in production, distribution and delivery. Institutionalized inefficiency, inadequate standards and lack of enforcement of standards have all created an environment where the substitution of lower grade or fraudulent products is easy to do and is now reported as widespread in the marketplace. This delivery of mislabelled products is not readily detected by consumers, particularly in new consumer markets. In recent years, the trade has seen a progressive overall reduction in olive oil prices despite a global trend to higher food pricing. Lower-margin wholesale and retail sales models have reduced the incentive for olive oil producers and the supply chain to react to increasing demand for quality. This presentation will summarize the current critical status of the olive oil industry. It will introduce options for standards, technology, policy and action by the supply chain that can address this situation for producers and consumers as well as re-create profitability in the sector. The current conditions in some global markets will also be discussed, as will the variations in regulatory reality in these markets.

### 2.20. Adulterations of Olive Oil

#### Mailer, R.J.; Ayton, J.

Olive Oil is considered the number one food product most at risk of fraud (Moore, J. *J. Food Sci*. 2012, *77*, R118). The high price of olive oil in relation to seed and other edible oils makes it a target for blending and substitution with cheaper products. Numerous studies in Europe, USA and Australia have revealed that the practice is widespread and more oils than not fail to meet international standards. Adulteration is an addition of another substance to a food item in order to increase the quantity of the food item, which may result in the loss of actual quality of the food item (Wikipedia). In fact, the term “fraud” is more appropriate to account for other cases of substitution and mislabelling. Olive oil may be sold as extra virgin olive oil but may be blended seed oil, refined olive oil or simply degraded oil, which has been subject to poor handling or long term storage, or both.

Despite the attempts of the International Olive Council and its highly proficient team of research chemists, olive traders continue to flood the market with poor quality products. This is due to the limited amount of testing by authorities and the absence of tests, which reveal oils which have degenerated through poor storage, age and temperature. Soft refining poses a problem in detecting oil that has been regenerated from decomposed olives and olive oil. The limits set for oil to restrict fraud often have negative effects in rejecting genuine olive oil, which varies simply due to cultivar or seasonal conditions.

### 2.21. Nutritional Aspects of Extra Virgin Olive Oil Compared with other Grades

#### Guillaume, C.

There are major health benefits associated with high quality Extra Virgin Olive Oil (EVOO). Extra Virgin Olive Oil contains high levels of natural antioxidants (polyphenols, squalene, tocopherols), which are beneficial for cardiovascular health (Fito, M., *et al*. *Atherosclerosis* 2005, *181*, 149–158) and anti-aging (Caramia, G., *et al*. *Minerva Pediatr*. 2008, *60*, 219–233). Those constituents also have been shown to help to reduce blood pressure and play an important role in protecting blood lipids from oxidative damage (Perez-Jimenez, F., *et al*. *Mol. Nutr. Food Res.* 2007, *51*, 1199). Extra Virgin olive oil is the only cooking oil that contains oleocanthal, which is a natural anti-inflammatory compound with a similar mode of action than ibuprofen. Vitamin E (α-tocopherol) found in Extra Virgin Olive Oil also plays a role in prevention of lipoprotein oxidation, reducing the risk of atherosclerosis. Plus, Extra Virgin Olive Oil does not contain *trans* fats—which are fats that raise your cholesterol level, increasing your risk of heart disease (Martinez-Gonzalez, M., *et al*. *Br. J. Nutr.* 2014, *112*, 48), the leading killer of men and women in Australia.

### 2.22. Status and Advances in Olive Oil Processing

#### Ravetti, L.

Virgin olive oil is the oily juice obtained from olives, which is separated from the other components of the fruit solely by mechanical means. When obtained by using appropriate processing techniques and from high quality, fresh fruit that have no defects or alterations and which are at the suitable ripeness stage, the oil has exceptional chemical and organoleptic characteristics. It is one of the few vegetable oils that can be consumed in its raw state, preserving its fatty acid composition and minor component content—which is extremely important for health and nutrition, particularly its vitamins and antioxidant content. Unfortunately, not all virgin olive oils produced in the world fulfil the above conditions. Significant amounts of this product will be refined as a result of their unpleasant organoleptic characteristics and/or high acidity and other chemical and physical parameters. Experience has shown that virgin olive oil spoilage occurs almost exclusively as a result of deficient fruit handling and poor processing methods. Olive oil represents approximately 2.0% of the total world oil and fat production by volume and 11.2% by value. This higher value is recognition of its unique organoleptic and chemical and physical characteristics. It is precisely due to those characteristics that virgin olive oil is considered a true fruit juice. Each step needs to be performed with the utmost care in order to produce the highest possible quality, Extra Virgin olive oil.

### 2.23. Olive Oil Consumption and Changes in Erythrocyte Fatty Acids and Lipids Following a Mediterranean Diet Intervention; Results from the MedLey Trial

#### Davis, C.R.; Bryan, J.; Hodgson, J.M.; Wilson, C.; Murphy, K.J.

The Mediterranean diet is a heart healthy diet. Our Mediterranean diet (MedDiet) for cognition and cardiovascular health in the elderly (MedLey) study is a randomised controlled dietary intervention where participants followed a MedDiet (*n* = 70) or continued their habitual diet (HabDiet) (*n* = 67) for six months. Fasting erythrocyte fatty acids and blood lipid were measured at baseline, three and six months. Weighed food records were administered at baseline, two and four months to quantify dietary intake. Linear mixed effect modelling was used to determine changes between groups over time for olive oil, lipid levels and erythrocyte fatty acids. Pearson’s correlations determined the relationship between olive oil intake, lipid levels and erythrocyte fatty acids.

Olive oil intake increased from 3 ± 5 to 30 ± 20 g/day (*p* < 0.001) in the MedDiet group, with no change in the HabDiet group (5 ± 7 to 4 ± 9 g/day). Per cent erythrocyte monounsaturated fat (MUFA) increased and saturated fat decreased in the MedDiet group compared to the HabDiet group (*p* < 0.05). Similar changes were observed for dietary intake of MUFA and saturated fat (between group differences *p* < 0.001). There was a positive correlation between erythrocyte MUFA and intake of olive oil at six months (*r* = 0.324, *p* < 0.05). There were negative correlations for total cholesterol and LDL cholesterol with total olive oil intake at six months (*r =* −0.253, *p* < 0.05 and *r* = −0.233, *p* < 0.05, respectively). Olive oil intake was negatively correlated with LDL (*r =* −0.233, *p* < 0.05).

Participants following a MedDiet substantially increased their intake of olive oil translating to a more favourable erythrocyte fatty acid profile for heart health.

### 2.24. Molecular Responses Underlying Metabolic Engineering of Oil in Plant Biomass

#### Divi, U.; Vanhercke, T.; Green, A.; Singh, S.; Petrie, J.

Oil or triacylglycerol (TAG) accumulates in plant seeds as a major renewable source of carbon for food, fuel and industrial feedstock. Supply of vegetable oils is in huge demand due to increasing world population and rising petroleum prices. Growing limitations on arable land and agricultural inputs mean it will be difficult to meet this demand with current oilseed-based production systems. Approaches to enhance oil content by engineering lipid pathways and genes in vegetative parts have gained significant attention as a way to intensify oil production. Such new oil production platforms would not only yield greater amount of oil for a given land area but also provide a way to more easily segregate bioeconomy traits such as unusual fatty acids away from food production. We previously reported the accumulation of up to 17% TAG (dry weight) in leaf tissue of *Nicotiana* species. This was achieved by combinatorial metabolic engineering in which we increased fatty acid biosynthesis (“Push”) by limited overexpression of the WRI1 transcription factor, increased TAG assembly (“Pull”) by expressing DGAT1, and encouraged oil body formation (“Packaging”) by expressing oleosin in plant leaves (Vanhercke T., *et al.* *FEBS* 2012, *587*, 364; Vanhercke, T., *et al.* *PBJ* 2014, *12*, 231.).

In this presentation, we will describe some of our second generation construct designs which have more than doubled the previously reported TAG content matching with some elite oilseed crop seed levels. We will also present preliminary data on transcriptome responses in high oil leaves compared to wild type leaves at different stages during plant development.

### 2.25. From Medieval to Space Age: High Throughput Assay for Determining Oil Phenotype Heterozygosity of Seed

#### Taylor, M.; Warden, A.; Okada, S.; Zhou, X.; Singh, S.; Wood, C.

Genetic screening of samples (plants, *etc.*) for specific genotypes can be a time consuming and labour extensive task. Here we describe a high throughput assay for quickly determining the fatty acid composition of oil from individual seeds, using robotics, novel lipidomics applications and bioinformatic analysis pipelines. By taking advantage of the unique properties of triacylglycerol (TAG) species, we have simplified our lipidomics techniques to semi-quantitatively determine TAG species and their sum fatty acids in a three minute run. In contrast to the high end technology, we used sheer brute force for seed processing and developed a stainless steel 96-well plate seed press which requires almost two tonnes of pressure to crush the seed. Oils were extracted and robotically diluted into 384 well plates and run continuously in the mass spectrometer. Using these techniques we were able to quickly and accurately determine the lipid composition of 2400 samples in a one-week period. Furthermore, a unique data analysis stream was developed for MS/MS data collection and bioinformatics analysis to convert TAG species into fatty acid composition and able to quickly and reproducibly determine the per cent fatty acid in oil. Using these techniques we were able to determine the difference of species producing 88% and 93% oleic acid and were able to identify whether individual plants were homozygous or heterozygous for the required genotype. These results were validated using FAME. High throughput techniques can be developed for surveying large populations, including plant seeds, human samples, for genetic or phenotypic screening.

### 2.26. Versatility of Inexpensive Lipoxygenase Enzyme from Soybean Flour

#### Tu, H.T.; Dobson, P.; Barrow, C.J.; Adcock, J.L.

Soybean flour is an inexpensive and natural source of lipoxygenase (LOX) enzymes which catalyse the controlled oxidation of LC-PUFAs. Soybean flour contains two types of active LOX—LOX-1 and LOX-2. LOX-1 can react with free fatty acids only, whereas type-2 LOX can oxygenate both free and esterified fatty acids. In this work, soybean flour was used as a LOX source (sfLOX) for the synthesis of anti-inflammatory compounds, resolvin D5 (RvD5) and protectin DX (PDX), from docosahexaenoic acid (DHA, C22:6n-3). The potential of sfLOX to react directly with trilinolein (TL) without hydrolysis was also investigated. The sfLOX-DHA and sfLOX-TL reactions were optimised to maximise the yield of di-hydroxylated compounds and mono-hydroxylated compounds, respectively. A range of analytical techniques, including GC-MS and chiral HPLC, were used to characterise the sfLOX-DHA products. The results showed that sfLOX had approximately the same efficiency in making two di-hydroxy compounds from DHA, 10*S*,17*S*-dihydroxydocosahexa-4*Z*,7*Z*,11*E*,13*Z*,15*E*,19*Z*-enoic acid (PDX) and 7*S*,17*S*-dihydroxydocosahexa-4*Z*,8*E*,10*Z*,13*Z*,15*E*,19*Z*-enoic acid (RvD5), as a commercially available soybean 15-sLOX-1 enzyme preparation (Dobson, E.P., *et al*. *J. Lipid Res*. 2013, *54*, 1439–1447). Unlike 15-sLOX-1, sfLOX was found to react with TL generating a range of mono-, di- and tri-hydroxylated triacylglycerols, which has a number of potential applications in the selective modification of PUFAs in complex lipid mixtures. This work demonstrated that soybean flour is a more versatile LOX source than commercial 15-sLOX-1 for certain applications. It could be used as a more economical enzyme source for the synthesis of bioactive compounds for the nutraceutical industry, and for reaction with esterified LC-PUFAs.

### 2.27. Kelp Phospholipids

#### Vyssotski, M.; MacKenzie, A.; Scott, D.; Lagutin, K.

Edible brown algae have attracted interest as a source of beneficial allenic carotenoid fucoxanthin, and glyco- and phospholipids enriched in polyunsaturated fatty acids. Unlike green algae, brown algae contain no or little phosphatidylserine, possessing an unusual aminophospholipid instead. In 1994, the structure of this novel phospholipid was suggested as *N*-(1-carboxy-3-aminopropyl-3)-1,2-diacyl-sn-3-glycerophosphorylethanolamine, abbreviated as *N*-CAPE (Schmid, C.E., *et al*. *J. Plant Physiol*. 1994, *143*, 570–574). An alternative structure of that lipid was published a year later, this time as phosphatidyl-*O*-[*N*-(2-hydroxyethyl)glycine], abbreviated as PHEG (Eichenberger, W., *et al*. *J Plant Physiol.* 1995, *146*, 398). While the PHEG lipid is more commonly referred to in the recent literature, *N*-CAPE structure is still occasionally mentioned, thus causing confusion. When our routinely used technique of ^31^P-NMR analysis of phospholipids was applied to the samples of edible New Zealand brown algae, a number of signals corresponding to unidentified phosphorus-containing compounds were observed in total lipids. NI ESI QToF MS spectra confirmed the presence of more familiar phospholipids, and also suggested the presence of PHEG or its isomers. The structure of PHEG was confirmed by comparison with a synthetic standard. An unusual MS fragmentation pattern that was also observed prompted us to synthesise a number of possible candidates, and was found to resemble that of phosphatidylhydroxyethylmethylcarbamate. An unexpected outcome was the finding of ceramidephosphoinositol that has not been reported as occurring in brown algae. An uncommon arsenic-containing phospholipid has also been observed and its TLC behaviour studied, along with that of the newly synthesised lipids.

### 2.28. Evaluating Suitability of Bead Milling on Lipid Recovery from Marine Microalgae for Omega-3 Fatty Acid Extraction

#### Byreddy, A.R.; Barrow, C.J.; Puri, M.

Omega-3 fatty acids (particularly DHA and EPA) production from microalgae has gained a significant interest over recent years due to health benefits and also due to their ability to accumulate up to 30%–70% of total lipids (Gupta, A., *et al.* *Biochem. Eng. J.* 2013, *78*, 11). Microalgae can be used to produce bulk ingredients for applications in pharmaceuticals, cosmetics, nutraceuticals, animal feeds and functional food industries. However, selection of efficient microalgae species and suitable lipid extraction methods are important for commercial bioactive production (Byreddy, A., *et al.* *Marine Drugs* 2015, *13*, 5111). All valuable microbial components such as lipids, proteins, carotenoids and carbohydrates must be extracted and purified in downstream processes prior to their respective applications. This study investigated the development of suitable downstream processing techniques for microalgae lipid recovery, a step that is considered a substantial contributor to total costs in most biotechnological processes. Thraustochytrid cells were disrupted using bead mill for maximising lipid extraction. Various parameters such as bead size, residence time, chamber volume, number of cycles were optimised to maximise lipid extraction. The extracted oil was characterized and further employed for concentration of omega-3 fatty acids using lipase. At the time of presentation, percentage increase in total omega-3 fatty acid content will be discussed.

### 2.29. A New Wave in Separation: Megasonics for Enhancing Recovery of Oil from Oleaginous Materials

#### Juliano, P.; Augustin, M.A.

Megasonic separation of oil consists of the application of high frequency (>0.4 MHz to several MHz) acoustic standing waves to enhance oil removal from oleaginous materials. The standing wave field positions individual droplets or particles on pressure nodes or antinodes within the reactor, promoting rapid agglomeration or coalescence into larger entities. With the formation of larger droplets or clustering of discrete oil droplets, the separation of oil from the non-oil solids is facilitated upon gravity settling or upon centrifugation. We describe the journey from concept to commercialisation, which resulted in the enhanced recovery of crude palm oil during the milling process. These findings are being applied to assess the potential of megasonics for improving oil recovery from a range of other oleaginous materials including coconut meal, algal biomass and olive fruit. This presentation will provide an overview of the application of megasonics technology in the oil industry, focussing on its potential to enhance oil recovery from oleaginous materials using aqueous-based extraction processes.

### 2.30. Partially Hydrogenated Oils: The Basics and The Latest News

#### Inturrisi, L.; Priddy, L.

Based on studies linking the consumption of *trans* fatty acids (TFA) and an increased risk of coronary heart disease, leading health advocates recommend limiting consumption of industrially produced TFA (iTFA) in the diet. Since partially hydrogenated oils (PHOs) are considered the most significant source of iTFA in the diet, policy makers around the world are increasingly responding with regulatory actions aimed at limiting PHOs in foods. Recently, The US Food and Drug Administration’s (FDA) determined that as of 18 June 2018, PHOs are no longer generally recognized as safe (GRAS). This means that from the above date, PHOs no longer have approved regulatory status in the USA, unless FDA approves its use as a food additive. While the FDA’s decision has made headlines worldwide and the effect has been felt in Asia Pacific. Many companies are considering alternatives to PHOs. This presentation will discuss the technical basics of PHOs and the latest regulatory and customer developments pertaining to PHOs.

### 2.31. Oil Binding Capacity of Palm Oil Based Structural Fat for Margarine and Spreads

#### Kanagaratnam, S.; Sahri, M.M.; Hoque, M.E.; Spowage, A.

The oil binding capacity (OBC) of structural fats play a vital role in determining the amount of solid fats (saturated) required to form a stable solid structure in margarines and spreads. The OBC determines the liquid-solid balance in the blends, which in turn indicated the stability. This study evaluated the OBC of palm oil fractions (POFs) as structural fat in oils blends. POFs of IV30, 20, 14 and 12 were evaluated with soybean oil. Soybean oil to POFs blends with ratios of 97.5:2.5, 95.0:5.0, 92.5:7.5, 90.0:10.0 and 87.5:12.5 (*w*/*w*) were texturized and evaluated. The OBC and the solid fat content (SFC) profile of the blends were the main criteria to determine suitable blends. OBC in this study was defined as the ability of the texturized blends to resist the centrifugal force applied to distort the crystal structure and remain intact. POF IV30 was not an effective structural fat in evaluated blends as it was not able to provide stability of 95% and above within the scope of this study. The study concluded POFs IV20 had stability above 95% at blending ratios 87.5:12.5, and with POF IV14 and IV12 had stability above 95% at blending ratios of 90.0:10.0 and 87.5:12.5. These binary blends of soybean oil with PSF IV14 and IV12 were also able to provide acceptable SFC. The SFC recorded was 6% and 8% at 35 °C and 13% and 16% at 5 °C for 90.0:10.0 and 87.5:12.5, respectively. PSF IV20, IV14 and IV12 blends with soybean oil at specific blending ratios were able to perform as an effective structural fat.

### 2.32. Development of Super High Oleic Safflower Oil for Oleochemical Applications

#### Wood, C.; Liu, Q.; Taylor, M.; Okada, S.; Zhou, X.; Singh, S.; Green, A.

Oleic acid is a highly preferred raw material for a range of oleochemical products, including lubricant, and polymers and plasticizers. The current high oleic (HO) safflower oil containing 70%–80% oleic acid is suboptimal for such high value applications as the residual linoleic acid can be broken into undesirable short chain monomers which are difficult to remove. Microsomal oleoyl phosphatidylcholine desaturase (FAD2) is largely responsible for the ratio of oleic and linoleic acids, together of which accounts for more than 90% of total fatty acids in safflower seed oil. From safflower, we have isolated the largest FAD2 gene family in plants, with a staggering 11 members with distinct expression patterns and functionalities. Molecular characterisation of the current safflower HO germplasm revealed that a spontaneous mutation by a single nucleotide deletion in the coding region of FAD2-1 resulted in frame shift and function loss. On this HO genetic background, RNAi mediated gene silencing targeting the constitutively expressed FAD2-2 produced safflower seed oil containing 94% oleic acid, so called super high oleic (SHO) safflower oil. Oxidative stability testing revealed that SHO safflower oil was three times more stable than standard HO oils. Biochemical and agronomic performance of selected transgenic lines indicate a promising prospect for commercial production of oleic acid as a valuable raw material for the formulation of many hundreds of biodegradable lubricants and oleochemicals.

### 2.33. Surfactant Impact on Inhibition of Fat Nucleation and Crystal Growth in Palm Olein Based Cooking Oil

#### Forrest, B.

Palm based cooking oil is common in tropical and subtropical climates. One quality aspect of retail cooking oil is to stay liquid, transparent and crystal free throughout shelf life. To address this need, palm olein, particularly with IV > 58, is used. Nevertheless, palm olein can begin to crystallize, reducing oil clarity and saleability. This behaviour becomes more apparent with storage in air-conditioned stores and cooler climates and weather (temperatures below 18 °C). To address the problem, producers have several options available to them. Examples include fractionating to a higher iodine value, addition of low melting point oils and the addition of surfactants that interfere with crystal growth and nucleation. Sorbitan tristearate and polyglycerol esters are well known crystal inhibitors. More recently, we have discovered that stearoyl lactylates can also interfere with onset of crystallization in palm olein based cooking oils, both in their own right and by potentiating the effect of other crystal inhibitors. The capacity of emulsifier combinations to inhibit crystallization in a range of cooking oils containing palm olein across the temperature range 0 °C to 18 °C will be shown. Emulsifier dosages typically between 300 and 500 ppm produce a significant improvement in delay of precipitation of lipids in the cooking oil.

### 2.34. The Implications of BPA on Early Developmental Obesity

#### Yoganantharajah, P.; Ward, A.; Gibert, Y.

Bisphenol A (BPA) is a chemical compound commonly found in polycarbonate plastics; currently its involvement in lipid deposition and adipogenesis during embryogenesis is unknown. BPA has been banned by many countries, especially in the production of baby products but not in Australia. We investigated if BPA exposure at physiological doses; 10 μM causes increased lipid deposition and changes lipid species in zebrafish embryos. We also determined the effect on the expression of lipidogenic and adipogenic markers in developing zebrafish for; lipoprotein lipase, CCAT/enhancer-binding protein alpha and liver fatty acid binding protein. A significant increase in expression was observed in BPA exposed embryos in all genes studied. The quantity of lipid deposition was observed via Oil-Red-O staining (ORO), a stain that specifically binds to neutral lipids and triglycerides. We developed a new technique for quantifying ORO staining in zebrafish using optical density as previously no quantification method were available for ORO staining during embryogenesis. We demonstrated that in embryos exposed to BPA there was a two-fold increase in ORO staining compared to WT embryos. This indicates a significant increase in the deposition of neutral lipids and triglycerides. To measure the changes in lipid species, lipidomics analysis using tandem mass spectrometry was performed; we were able to analyse changes across 24 lipid classes in BPA exposed embryos. Since we are able to show that BPA has a substantial effect on lipid deposition and adipogenesis during embryogenesis, we believe its use in Australia should be reconsidered.

### 2.35. Lipid Soluble Green Tea Extract: A New Natural Flavouring with Excellent Oil-Stabilization and Antioxidative Properties in Food Emulsions and Frying Oils

#### Huang, S.; Ban, L.; Yuan, Q.; Schroeder, W.

Lipid Soluble Green Tea Extract (LSGT) is a tea polyphenol derivative obtained from the esterification of green tea extract (GTE) and palmitic acid. It widely exists in green tea leaves (Myers, R.A., *et al*. *J. Agric. Food Chem*. 2013, *61*, 11484). LSGT overcomes the solubility issue of GTE in oil/fats without much loss of the antioxidant activity. When applied to food emulsions including margarine, salad dressing and mayonnaise, LSGT showed much better anti-oxidative ability than other natural antioxidants like rosemary extract (RE) and mixed tocopherols (MT). In margarine, 250 ppm LSGT treatment stayed <5 meq/kg lipid peroxide value for the whole storage period while commercial positive controls (150 ppm BHA + BHT and 100 ppm MT) and RE treatments (500 and 1000 ppm) reached as high as 7–14 meq/kg. The analysis of secondary oxidative products also showed the same trend. LSGT was proved to be a much more effective natural flavouring in margarine. In salad dressing and mayonnaise, it was found that LSGT could extend their shelf life comparative to EDTA and longer than RE. It could be used as a substitute for EDTA. A series of LSGT-based blends were also screened to stabilize deep-frying oils. LSGT, in combination with RE and MT, decreased the total polar compound (threshold value 27%) from 24.9% to 15.0% for frying soybean oil and from 16.4% to 11.1% for frying palm oil after 40 frying circles. In conclusion, LSGT provided a natural alternative solution to extend the frying circle of deep-frying oils and therefore is a better cost effective option for frying food producers.

### 2.36. Monoacylglycerol Acyltransferase Expression in Arabidopsis thaliana Yields Higher Lipid Accumulation in Seeds

#### Tahchy, A.E.; Petrie, J.R.; Shrestha, P.; Vanhercke, T.; Singh, S.P.

Plant triacylglycerol (TAG) biosynthesis routes such as the Kennedy pathway do not include a monoacylglycerol acyltransferase (MGAT) step to catalyse the acylation of monoacylglycerol (MAG) to form diacylglycerol (DAG). Rather, DAG and TAG are synthesised *de novo* from glycerol-3-phosphate (G-3-P) by a series of three subsequent acylation reactions. This study describes the stable expression of the *Mus musculus* MGAT2 in *Arabidopsis thaliana* that significantly increased lipid content in seed by 1.32 fold over the control. Based on *in vitro* seed lysate assays with labelled sn2-MAG and unlabelled oleoyl-CoA, 1.9 to 3.9 fold of radiolabelled DAG were produced. The radiolabelled DAG levels observed suggest that the transgenic MGAT activity can result in DAG synthesis by salvaging the MAG that is a product of TAG breakdown creating a route that is independent and complementary to the endogenous Kennedy pathway and other glycerolipid synthesis routes.

### 2.37. Detecting Lipid Oxidation in Pet Food

#### Learey, J.J.; Adcock, J.L.; Crawford, S.; Barrow, C.J.

Omega-3 and omega-6 fatty acids are added to pet food due to their health benefits, however these ingredients are highly unstable and undergo minor oxidation leading to off-odours and flavours even at very low levels. This study aimed to develop simple and sensitive methods capable of monitoring these lipid oxidation processes through the use of chemiluminescence and electron paramagnetic resonance (EPR). The chemiluminescence reagent luminol has previously been demonstrated as an alternative to the current colorimetric tests including the peroxide value (Rolewski, P., *et al*. *Food Res. Int.* 2009, *42*, 165). In this work we demonstrate the potential of another chemiluminescence reagent, manganese (IV), for detecting lipid oxidation products. The use of EPR has allowed for changes in the intensity of radical intermediates formed during this degradation process to also be analysed. While the presence of antioxidants has been shown to affect these results, both techniques offer advantages over currently employed methods in terms of their simplicity and sensitivity.

### 2.38. 4-Hydroxy-naphthalimide Esters: New Substrates for the Fluorometric Detection and Characterisation of Lipase and Esterase Activity

#### Nalder, T.D.; Ashton, T.D.; Marshall, S.; Pfeffer, F.M.; Barrow, C.J.

New fluorescent substrates have been synthesised and used to measure the activity of lipases and esterases. The 4-hydroxy-naphthalimide fluorophore was esterified with short, medium and long chain fatty acids, which were chemically characterised, and their properties investigated for their suitability for use in enzyme assays. The substrates were found to be relatively stable under the conditions of the assay and underwent minimal spontaneous hydrolysis. A simple and rapid fluorometric assay methodology was developed and the substrates were used to analyse the activity of four commercially available enzymes; lipase B from *Candida antarctica* (CaLB), *Thermomyces lanuginosa* lipase (TlL), *Candida rugosa* lipase (CrL) and esterase from *Pseudomonas fluorescens* (PfE). Further to this the substrates have been implemented in the kinetic profiling of these enzymes. This assay methodology was found to be highly sensitive and allowed for the determination of fundamental differences in activity between lipases and esterases. These new 4-hydroxy-naphthalimide esters provide an alternate fluorescent method from established coumarin-based fluorophores, possessing distinctly different excitation and emission maxima.

### 2.39. Nutritional Characterisation of Australian Marine Macroalgae

#### Skrzypczyk, V.M.; Hermon, K.M.; Norambuena, F.; Turchini, G.M.; Bellgrove, A.

Algal production accounts for a total annual value of US$ 6 billion globally. Temperate southern Australian waters are considered a “biodiversity hotspot”, because of the high diversity and endemism within taxonomic groups (Phillips, J., *et al*. *Biodivers. Conserv*. 2001, *10*, 9). Whilst similar algal species are abundantly harvested commercially elsewhere worldwide, very little is known about southern Australian algae and their commercial exploitation is fundamentally nil. Algae are known to contain nutritionally beneficial compounds that are associated with a number of potential health benefits. Unsaturated fatty acids constitute >70% of the total fatty acids in marine algae, with lipid content varying from <1% to 11%. Despite the low total lipid content, a large proportion consists of polyunsaturated fatty acids (PUFA) thus making seaweed a healthy low fat food. This study aimed to determine the nutritional profiles of nine local marine macro-algae, which were compared to four Japanese species traditionally and abundantly consumed. The nutritional parameters investigated included fatty acid and proximate composition (total lipid, protein, moisture, ash, and crude fibre), to identify the overall nutritional values of different species. Lipid, omega-3 DHA and total FA content was highest in Australian species, whilst Japanese species generally contained higher amounts of protein, fibre, and omega-3 EPA. *Codium galeatum* met the recommended omega-3 ALA serving guidelines, with all species excluding *Phyllospora comosa, Durvillaea potatorum* and *Polysiphonia decipiens* classed as being a source of omega-3 fatty acids (Turchini, G., *et al*. *Crit. Rev. Food Sci*. 2012, *52*, 9).

### 2.40. Lipase-Catalysed Incorporation of EPA into Emu Oil: Formation and Characterisation of a New Structured Lipid

#### Akanbi, T.O.; Barrow, C.J.

Partial hydrolysis of emu oil (EMO) was performed using *Thermomyces lanuginosus* lipase to remove some shorter chain fatty acids. Then eicosapentaenoic acid (EPA) was incorporated into the modified emu oil using either Lipozyme RMIM or Lipozyme TLIM to produce new EPA enriched structured lipids. Using isooctane as a reaction solvent increased the level of EPA incorporation, which was higher with RMIM than with TLIM. Positional distribution of EPA in the modified oil, as determined by ^13^C nuclear magnetic resonance (NMR) spectroscopy, showed that TLIM incorporated EPA mainly into sn-1,3 position, whereas RMIM incorporated EPA in both sn-1,3 positions sn-2 at an approximate statistical ratio of 2.5:1. However, regardless of the positional distribution of EPA, the resulting structured lipids were less oxidatively stable than EMO, as measured by conjugated dienes (CD) and thiobarbituric acid reactive substances (TBARS) tests (Akanbi and Barrow JFF 2014—In Press).

### 2.41. Inhibitory Role of Krill Oil in the Proliferation of Colorectal Cancer Cells (HCT-15)

#### Jayathilake, A.; Senior, P.; Su, X.

Colorectal cancer (CRC) is the fourth leading cause of cancer death in the world. Combination treatment for CRC has not improved prognosis effectively. Due to the toxicity of available chemotherapy effective nutraceutic agents should be explored. Omega-3 polyunsaturated fatty acids, mainly eicosapentaenoic acid (EPA) and docosahexaenoic acid (DHA) are known to have many health benefits including inhibition of cancer progression. Krill oil has been suggested to have a higher bioavailability than fish oil due to its unique chemical composition. This study assesses the effects of krill oil, in comparison with fish oil, on the proliferation of human CRC. HCT-15 cells were seeded in culture medium of 100 μL in 96-well plates at the density of 10^4^ cells/well. Free fatty acids extracted from krill oil and fish oil were dissolved in 1% DMSO. The concentration of treatment solutions ranged from 0.025% to 0.4%. Commercially available EPA and DHA were dissolved in 0.1% ethanol and the final treatment concentrations were 50 μM, 100 μM and 200 μM. Cell proliferation after 48 h of treatment was assessed via WST-1 assay. Krill oil significantly inhibited cancer cell growth at concentrations of 0.1%–0.4%. However, no changes were observed at the lower concentration (0.05%). Fish oil showed significant inhibition on cell growth at concentrations of 0.05%–0.4%. Similarly EPA also showed marked inhibition at 200 μM. These results suggest that krill oil and fish oil may be useful as potential therapeutic agents for CRC treatment. The inhibitory effects of the marine oils on cell proliferation maybe attributed to their EPA. 

### 2.42. Postprandial Lipids Response to Krill Oil Supplementation in Healthy Women

#### Sung, H.; Sinclair, A.J.; Lewandowski, P.; Su, X.Q.

Krill oil (KO) as an alternative source of long chain n-3 polyunsaturated fatty acids (LC n-3 PUFAs) has been suggested to have higher bioavailability of EPA and DHA compared with fish oil. There are limited reports on the postprandial effects of KO. This study investigates the impacts of supplementation with KO and fish oil (FO) in comparison with olive oil (OO) on plasma lipid profiles in the postprandial state. The study was a randomised cross-over design. Test meals with three oils supplements (KO, FO or OO) were randomly provided on each study day with seven days wash-out period between. The test meal consisted of 150 g of fresh mashed potato and 15 g of olive oil. 5 g of supplemented oils were mixed with the test meal. Blood samples were collected at the baseline and post intervention on hourly basis for 5 h. Blood lipids were analysed using the auto lipid analyser. Postprandial changes in the parameters over time between the treatments were assessed using the SPSS package. *p* < 0.05 was considered statistically different. There were no significant difference in the absorption of triglycerides and cholesterol in either chylomicron or plasma between FO and KO. In addition no significant differences were observed between the treatment oils and olive oil. Since KO contains omega-3 fatty acids mainly in phospholipids, the omega-3 profile in postprandial plasma phospholipids should be examined (Linderborg, K.M*.*, *et al*. *PLEFA* 2013, *88*, 313–319).

### 2.43. Utilisation of Industrial Wastes by Thraustochytrids for Production of Lipids

#### Chang, K.L.; Dumsday, G.; Mansour, P.; Blackburn, S.I.; Nichols, P.D.

The potential production of biofuel and high-value lipids from microalgae is of intense interest globally. Thraustochytrids are heterotrophic protists, which are characterised by their capacity to produce health-benefitting omega-3 long-chain (≥C20) polyunsaturated fatty acids (ω3 LC-PUFA), including 22:6ω3 (docosahexaenoic acid, DHA). Cultivation of thraustochytrids for biofuels production using industrial wastes as a feedstock can also provide a solution to the current situation of limited biomass feedstock availability; this issue is expected to restrict industry uptake of the development and application of transportation biofuels in Australia. The co-production of high-value lipids during biofuel production is desirable both from a diversified product perspective, to add greater value to the production process, and to improve process economics. We examined whether Australian industrial waste can be used as carbon and nitrogen sources by recently isolated endemic thraustochytrids for the production of biofuel and high-value lipids. Lipid and growth profiles will be presented for thraustochytrids grown in industrial wastes such as crude glycerol, soy pulp and protein meals. The crude glycerol was not pre-treated prior to addition to the culture medium, with an overall aim of avoiding or minimizing unnecessary processing costs. Impurities in the crude glycerol hindered growth of thraustochytrids, with maximum yield of 9 g/L dry cell weight and 48% DHA of total fatty acids achieved at four-days under the shake-flask conditions used. Although our results indicate further research is required, we show that heterotrophic cultivation of thraustochytrids in industrial wastes offers considerable potential for commercial production of biofuels and other high-value lipids.

## Author Affiliations

- Jayathilake, A. Centre for Chronic Disease, Victoria University, Melbourne, Australia
- Bellgrove, A. Centre for Integrative Ecology, Deakin University, Geelong, Australia
- Ward, A. Deakin University, Geelong, Australia
- Green, A. CSIRO Food and Nutrition, Canberra, ACT, Australia
- Sinclair, A.J. Deakin University, Geelong, Australia
- MacKenzie, A. Callaghan Innovation, Wellington, New Zealand
- Spowage, A. University of Nottingham Malaysia Campus, Semenyih, Selangor, Malaysia
- Warden, A. CSIRO Land and Water, Canberra, ACT, Australia
- Tahchy, A.E. CSIRO Agriculture National Research Flagship & Food and Nutrition Research Flagship, Canberra, ACT, Australia
- Byreddy, A.R. Centre for Chemistry and Biotechnology, Deakin University, Geelong, Australia
- Forrest, B. Danisco Australia Pty Ltd., M. Nielsen, DuPont NHIB Denmark
- Adhikari, B. RMIT University, Melbourne, Australia
- Wang, B. Deakin University, Geelong, Australia
- Wilson, C. Flinders University, Adelaide, Australia
- Guillaume, C. Modern Olives Laboratory Services, Lara, Australia
- Barrow, C.J. Centre for Chemistry and Biotechnology, Deakin University, Geelong, Australia
- Davis, C.R. University of South Australia, Adelaide, Australia
- Wood, C. CSIRO Agriculture, Canberra, ACT, Australia
- Ying, D. CSIRO Food and Nutrition Flagship, Canberra, ACT, Australia
- Scott, D. Callaghan Innovation, Wellington, New Zealand
- Norambuena, F. Centre for Chemistry and Biotechnology, Deakin University, Geelong, Australia
- Pfeffer, F.M. Deakin University, Geelong, Australia
- Dumsday, G. CSIRO Manufacturing, Clayton, Victoria, Australia
- Turchini, G.M. Centre for Chemistry and Biotechnology, Deakin University, Geelong, Australia
- Tu, H.T. Centre for Chemistry and Biotechnology, Deakin University Geelong, Australia
- Sung, H. Victoria University, Melbourne, Australia
- Adcock, J.L. Centre for Chemistry and Biotechnology, Deakin University, Geelong, Australia
- Petrie, J.R. CSIRO Agriculture National Research Flagship & Food and Nutrition Research Flagship, Canberra, ACT, Australia
- Ayton, J. NSW Department of Primary Industries, Wagga Wagga, NSW, Australia
- Bryan, J. University of South Australia, Adelaide, Australia
- Learey, J.J. Centre for Chemistry and Biotechnology, Deakin University, Geelong, Australia
- Hodgson, J.M. University of Western Australia, Perth, Australia
- Hermon, K. School of Life and Environmental Sciences, Deakin University, Warrnambool, Australia
- Murphy, K.J. University of South Australia, Adelaide, Australia
- Hermon, K.M. Centre for Chemistry and Biotechnology, Deakin University, Geelong, Australia
- Chang, K.L. CSIRO Future Science Platforms and CSIRO Oceans and Atmosphere, Australia
- Lagutin, K. Callaghan Innovation, Wellington, New Zealand
- Ban, L. Kemin Food Technologies, USA
- Eyres, L. Chairman of the NZIC Oils and Fats Specialist Group, director of ECG consultancy, Warkworth, New Zealand
- Ravetti, L. Modern Olive, Lara, Australia
- Priddy, L. Cargill Refined Oils Asia Pacific, Australia
- Inturrisi, L. Technical Services Director, Cargill Refined Oils Asia Pacific, Australia
- Sanguansri, L. CSIRO Food and Nutrition Flagship, Canberra, ACT, Australia
- Hoque, M.E. University of Nottingham Malaysia Campus, Semenyih, Selangor, Malaysia
- Abeywardena, M. CSIRO, Adelaide, Australia
- Devine, M. Nuseed Pty Ltd., Australia
- Noakes, M. CSIRO Food and Nutrition Flagship, Canberra, ACT, Australia
- Augustin, M.A. CSIRO Food and Nutrition Flagship, Canberra, ACT, Australia
- Miller, M.R. Cawthron Institute, Nelson, New Zealand
- Taylor, M. CSIRO Land and Water, Canberra, ACT, Australia
- Vyssotski, M. Callaghan Innovation, Wellington, New Zealand
- Sahri, M.M. Malaysian Palm Oil Board, Kuala Lumpur, Malaysia
- Puri, M. Centre for Chemistry and Biotechnology, Deakin University, Geelong, Australia
- Joyce, N. New Zealand Institute for Plant & Food Research, Nelson, New Zealand
- Hoem, N. Aker BioMarine, Oslo, Norway
- Juliano, P. CSIRO, Melbourne, Australia
- Lewandowski, P. Deakin University, Geelong, Australia
- Miller, P. Australian Olive Association, Australia
- Senior, P. Melbourne Medical School Western Campus, Western Centre for Health Research and Education, Melbourne, Australia
- Nichols, P.D. CSIRO Food & Nutrition, Oceans & Atmosphere Flagships, Hobart, Australia
- McLennan, P.L. Graduate School of Medicine, University of Wollongong, Wollongong, NSW, Australia
- Mansour, P. CSIRO Oceans and Atmosphere, Hobart Australia
- Dobson, P. Centre for Chemistry and Biotechnology, Deakin University, Geelong, Australia
- Yoganantharajah, P. Deakin University, Geelong, Australia
- Shrestha, P. CSIRO Agriculture National Research Flagship & Food and Nutrition Research Flagship, Canberra, ACT, Australia
- Pepe, S. Murdoch Children’s Research Institute, Royal Children’s Hospital, Melbourne, VIC, Australia
- Liu, Q. CSIRO Food and Nutrition Flagship, Canberra, ACT, Australia
- Yuan, Q. Kemin Food Technologies, Zhuhai, Guangdong, China
- Sambanthamurthi, R. Kajang, Selangor, Malaysian Palm Oil Board, Malaysia
- Mailer, R.J. Australian Oils Research, NSW, Australia
- Ghasemifard, S. School of Medicine, Deakin University, Geelong, Australia
- Huang, S. Kemin Food Technologies, Zhuhai, Guangdong, China
- Okada, S. CSIRO Land and Water, Canberra, ACT, Australia
- Crawford, S. Killara, NSW, Kemin, Australia
- Kanagaratnam, S. Malaysian Palm Oil Board and University of Nottingham Malaysia Campus, Semenyih, Selangor, Malaysia
- Singh, S.P. CSIRO Agriculture National Research Flagship & Food and Nutrition Research Flagship, Canberra, ACT, Australia
- Blackburn, S.I. CSIRO National Facilities and Collections, Hobart, Australia
- Marshall, S.N. New Zealand Institute for Plant & Food Research, Nelson, New Zealand
- Akanbi, T.O. Deakin University, Geelong, Australia
- Sanders, T. King’s College, London, United Kingdom
- Vanhercke, T. CSIRO Agriculture National Research Flagship & Food and Nutrition Research Flagship, Canberra, ACT, Australia
- Nalder, T.D. Centre for Chemistry and Biotechnology, Deakin University, Geelong, Australia; New Zealand Institute for Plant & Food Research, Nelson, New Zealand
- Walsh, T.R. Deakin University, Geelong, Australia
- Ashton, T.D. Deakin University, Geelong, Australia
- Lockett, T. CSIRO Food & Nutrition Flagship, Canberra, ACT, Australia
- Divi, U. CSIRO Food, Nutrition, Canberra, ACT, Australia
- Skrzypczyk, V.M. Centre for Integrative Ecology, Deakin University, Geelong, Australia
- Stonehouse, W. CSIRO Food & Nutrition Flagship, Canberra, ACT, Australia
- Schroeder, W. Kemin Food Technologies, Des Moines, IA, USA
- Su, X. Centre for Chronic Disease, Victoria University, Melbourne, Australia
- Zhou, X. CSIRO Agriculture, Canberra, ACT, Australia
- Gibert, Y. Deakin University, Geelong, Australia

